# A machine learning framework for genotyping the structural variations with copy number variant

**DOI:** 10.1186/s12920-020-00733-w

**Published:** 2020-08-27

**Authors:** Tian Zheng, Xiaoyan Zhu, Xuanping Zhang, Zhongmeng Zhao, Xin Yi, Jiayin Wang, Hongle Li

**Affiliations:** 1 School of Computer Science and Technology, Xi’an Jiaotong University, Xi’an, 710049 China; 2 Geneplus-Beijing, Beijing, 102206 China; 3 Department of Molecular Pathology, Henan Cancer Hospital, The Affiliated Cancer Hospital of Zhengzhou University, Zhengzhou, 450003 China

**Keywords:** Cancer genomics, NGS data analysis, Genotyping structural variation, Copy number variant, Multiclass relevance vector machine

## Abstract

**Background:**

Genotyping of structural variation is an important computational problem in next generation sequence data analysis. However, in cancer genomes, the copy number variant(CNV) often coexists with other types of structural variations which significantly reduces the accuracy of the existing genotype methods. The bias on sequencing coverage and variant allelic frequency can be observed on a CNV region, which leads to the genotyping approaches that misinterpret the heterozygote as a homozygote. Furthermore, other data signals such as split mapped read, abnormal read will also be misjudged because of the CNV. Therefore, genotyping the structural variations with CNV is a complicated computational problem which should consider multiple features and their interactions.

**Methods:**

Here we proposed a computational method for genotyping indels in the CNV region, which introduced a machine learning framework to comprehensively incorporate a set of data features and their interactions. We extracted fifteen kinds of classification features as input and different from the traditional genotyping problem, here the structure of variant may fall into types of normal homozygote, homozygous variant, heterozygous variant without CNV, heterozygous variant with a CNV on the mutated haplotype, and heterozygous variant with a CNV on the wild haplotype. The Multiclass Relevance Vector Machine (M-RVM) was used as a machine learning framework combined with the distribution characteristics of the features.

**Results:**

We applied the proposed method to both simulated and real data, and compared it with the existing popular softwares include *Gindel, Facets, GATK*, and also compared with other machine learning cores: *Support Vector Machine, Lanrange-SVM with OVO multiple classification, Naïve Bayes* and *BP Neural Network*. The results demonstrated that the proposed method outperforms others on accuracy, stability and efficiency.

**Conclusion:**

This work shows that the genotyping of structural variations on the CNV region cannot be solved as a traditional genotyping problem. More features should be used to efficiently complete the five-category task. According to the result, the proposed method can be a practical algorithm to correct genotype structural variations with CNV on the next generation sequence data. The source codes have been uploaded at https://github.com/TrinaZ/Mixgenotypefor academic usage only.

## Background

Structural variations(SVs) generally refer to cyto-genetically visible and submicroscopic variants, including insertion, deletion, inversion, copy number variant and etc [1, 2]. The genotype of SVs, also known as genotype analysis, is a technique to determine whether the structural variation is heterozygous or homozygous [3]. Obtaining the accurate genotypes of SVs can be widely used in downstream analysis, such as imputing genotypes [4], estimating genomic diversity [5], calculating linkage disequilibrium [6] and clinical practices including disease diagnosis [7], treatment management [8] and drug design [9].

Traditional methods used genomic chips to detect structural variations and their genotypes. In recent decades, with the development of the next generation sequence technology, sequencing data analysis has replaced the microarray. The existing methods often extract the data signals as features from the sequencing data and use these features to further estimate the genotypes. According to the strategies of incorporating the features, the existing methods can be divided into three categories, the first often rely on the overlapping points and breakpoints, such as *Pindel-C* [10]. The second category, include *piCALL* [11] and *MATE-CELEVER* [12], incorporate the features based on the Bayesian framework, and the third category, include *Gindel* [13] and *CIGenotyper* [14], adapt the machine learning models. Nevertheless, these methods do not take the effect of the copy number variant (CNV) into account and suffer an accuracy loss for tumor sequencing data. CNV is a kind of common structural variant that widely exists in cancer genome, which plays an important role in cancerization [15], recurrence [16], metastasis [17], drug resistance [18], and is associated with clinical diagnosis [19] and antipsychotic drugs [18]. Recent studies reported that a CNV often combines with other structural variations [20, 21], resulting in heterozygous variant being misjudged as homozygote, which seriously affects the accuracy of genotyping SVs on tumor data.

**Fig. 1:**
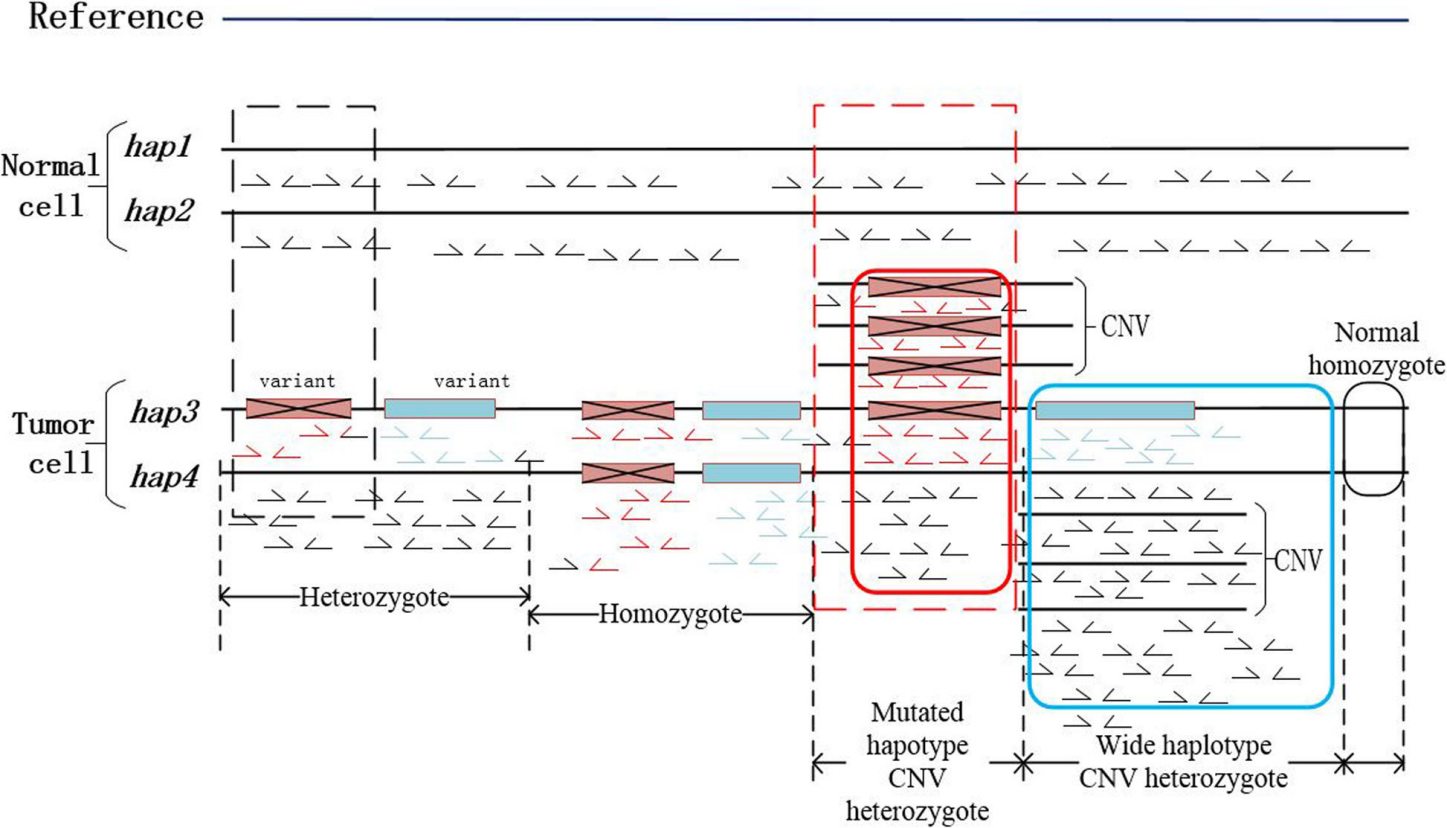
Genotype classification. The red rectangle indicates the deletion variant and the blue rectangle indicates the insertion variant. The black dotted rectangle indicates that the VAF of the CNV-free region has changed due to the purity of the tumor, the VAF is diluted by tumor purity and the coverage is same as the normal region. The red dotted rectangle indicates that the VAF and the coverage are both changed in the mutated haplotype CNV heterozygote region, where if the coverage of mutated haplotype equals to the wild haplotype, the former features can hardly distinguish the G3 and the G1

Specifically, to further investigate this computational problem, each human gene has two copies of the same haplotype and the CNV amplifies a one-sided signal, which causing the expression rate of the two gene copies deviated from 1:1 [22]. When a CNV appears on the haplotype harboring the variant, the number of reads in the mutated region presents multiple times more than that of the normal region, which may lead an existing method misclassifies a heterozygote to a homozygous variant, as shown by the red rectangle in Fig. 1. On the other hand, when a CNV appears on the wild haplotype, more reads mapped to the normal region will be observed and the signal of the variant side will be concealed, causing a heterozygous variant be misjudged as a non-variant homozygote, as shown by the blue rectangle in Fig. 1. Furthermore, these problems will be complicated by other data signals in cancer sequencing data. To name a few, all the tumor sample have the problem of purity and may cause the bias on data signals as shown by the black rectangle in Fig. 1, which is quite similar with the bias caused by CNV and may also be contributed by clonal structure. The tumor purity may change the variant allelic frequency (VAF) while may not contribute to the increase of coverage, if the purity causes the coverage of the normal hapolotper to be equal to the coverage of the mutated haplotype, a heterozygous variant with mutated haplotype CNV will be misjudged as a normal heterozygous variant, as shown by the red dotted rectangle in Fig. 1. Consequently, we have to further consider other data signals such as coverage, split mapped reads, read depth, extended read depth and their interactions to solve this problem. Moreover, recent studies reported that CNV is often combined with adjacent Single-Nucleotide Variants, which is one signal in detecting the CNV region and should also be considered. Other examples will not be repeated here, but it should be pointed out that other methods do not consider multiple features and their interactions may lead to computation exploration. It’s inefficient to either consider single feature or use a Bayesian framework, while the machine learning framework is the most effective choice in this case with limited training samples.

In conclusion, the existing genotyping methods may suffer accuracy loss in processing the tumor sequencing data regardless the CNV, and the genotyping problem should be considered comprehensively as shown in Fig. 1. The computation problem in this case is complicated into a five category problem which a structural variant may fall into one of the five category: normal homozygote (N), homozygous variant (G1), heterozygous variant without CNV (G2), heterozygous variant with a CNV on the mutated haplotype (G3), and heterozygous variant with a CNV on the wild haplotype(G4). We proposed 15 features to train the model based on extensive research and chose the Multiclass Relevance Vector Machine(M-RVM) as the machine learning framework based on the data analysis. We applied the proposed method on both simulated and real data, compared it with the existing popular softwares include *Gindel* [13], *Facets* [23], *GATK* [24], and also compared with other machine learning cores, *Support Vector Machine, Lanrange-SVM with OVO multiple classification, Naïve Bayes* and *BP Neural Network*. The results showed that the average of our method accuracy is 83% (±2%) on simulated data and 88.2% (±15%) on real data, while the classification accuracies of other methods are around 65% on simulated data and 75% on real data with more than 20% range at the same condition, which demonstrated that the proposed method outperforms others on accuracy, stability and efficiency.

## Methods

### The genotype representations

Considering the genotyping problem, we defined five classes of genotypes: normal homozygote (N), homozygous variant (G1), heterozygous variant without CNV (G2), heterozygous variant with a CNV on the mutated haplotype (G3), and heterozygous variant with a CNV on the wild haplotype (G4). Each category is represented by vectors [0,0,0,0,1]^*T*^, [0,0,0,1,0]^*T*^, [0,0,1,0,0]^*T*^, [0,1,0,0,0]^*T*^, [1,0,0,0,0]^*T*^, respectively, which is one of the output vectors of M-RVM and the other output is their probability [*p*_*N*_,*p*_*G*1_,*p*_*G*2_,*p*_*G*3_,*p*_*G*4_], where *p*_*N*_+*p*_*G*1_+*p*_*G*2_+*p*_*G*3_+*p*_*G*4_=1 and *p*_*N*_,*p*_*G*1_,*p*_*G*2_,*p*_*G*3_,*p*_*G*4_ represent the probabilities of the state *N*, *G*1, *G*2, *G*3 and *G*4, respectively. Note that the index set of the output vector is *I*={*N*,*G*1,*G*2,*G*3,*G*4}, and M-RVM finally output the genotype with the highest probability.

### Input features extraction

As mentioned above, to accurately classify genotypes has to incorporate multiple features which are extracted from the sequence data with the Standard Alignment/Map (SAM) or Binary Alignment/Map (BAM) format and list of candidate calls in the Variant Call Format (VCF). There are fifteen features considered in this approach as listed in Table 1. Details are discussed as follow.

**Table 1:** Features description

Description of Features		
1	Abnormal read	The number of abnormal insert size read pairs
2	Normal read	The number of normal insert size read pairs
3	Incompletely mapped reads	The number of incompletely mapped read pairs
4	Fully mapped read	The number of read pairs that can fully mapped to the reference
5	Split mapped read	The number of split mapped reads
6	Single mapped read	The number of single mapped reads
7	Unmapped read	The number of the mutated region reads which comes from the Samtools
8	Mapping qualities	Sum of mapping qualities of anchor reads
9	Read depth	The read depth of the mutated region
10	Weighted read depth	The read depth of the mutated region weighted by mapping qualities
11	Extended weighted read depth	Extended the weighted read depth to up down 100 bps each
12	Affected read	The number of affected reads
13	Variant length	The length of structural variant
14	Direction 1	The number of reads which clipping from 5’ during initial mapping
15	Direction 2	The number of reads which clipping from 3’ during initial mapping

#### Features based on the length of SV and the insert size relation

**Fig. 2:**
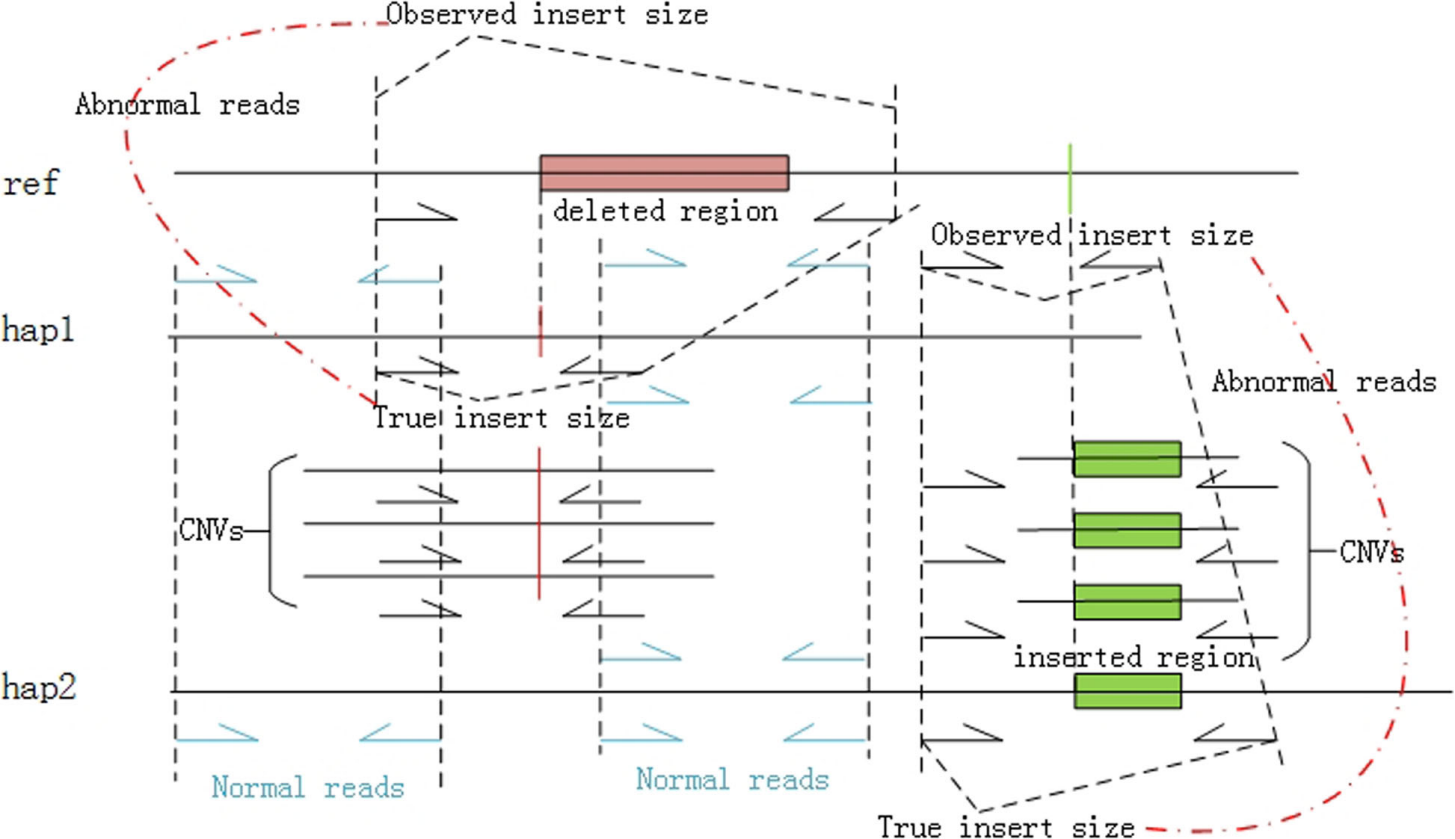
Features based on the insert size. The red rectangle indicates the deletion, and the green portion indicates the insertion variant. When the deletion occurs, the observed insert size is larger than the true insert size, while the observed insert size is shorter than the true insert size when harboring the insertion variant, which are all counted as the abnormal reads

The insert size is the length of template captured by the sequencer and the DNA fragments are expected to follow the normal distribution around insert size in paired-end sequence [13]. We set *μ* as the mean library insert size and *σ* as its standard deviation, which can be either specified by users or calculated from the given BAM/SAM file. We define the normal read as the read whose insert size located in the *μ*±3*σ* range, and position located in the mutated region. Accordingly, the read whose insert size deviates from normal distribution, exceeding the range of *μ*±3*σ*, and position located in the mutated region is defined as the abnormal read. Consequently, we extract the numbers of normal reads and abnormal reads as two features as shown in Fig. 2.

When we consider the normal structural variant, the number of abnormal read is an important index to detect the indel. For example, large deletions may introduce the abnormal reads with extremely large insert size, and large insertions may introduce the abnormal reads with extremely short insert size. Furthermore, in the CNV region, this is more complicated. For a heterozygous variant without consideration other factors, the ratio of normal read to abnormal read is roughly close to 1:1, when a CNV exists in the mutated haplotype and the copy number equals to *n*, the ratio is close to *n*:1, and when the CNV exists in the wild haplotype of a heterozygous variant, the ratio is close to 1:*n*. Thus, different from the existing approach, the number of abnormal reads and normal reads should be considered at the same time. In addition, if the insertion is extremely longer than the insert size, the variant may not introduce the abnormal reads, while if the insertion is shorter than the insert size, the abnormal reads will be multiplied. Accordingly, the length of structural variant (*L*) should also be considered as a feature, which can reflect the information about variant itself and indicate the difference in the relationship between the variant and the reads.

In practice, each row of the VCF file corresponds to a variant and indicates their matching positions respectively. Since the read length is 100 bps, we extract the second and the eighth columns, POS and End, from the VCF file, calculate the length of |POS-End | and record it as the *L*, and define the [POS-100, End+100] interval as the mutated region for each variant; Then select the row of the BAM/SAM file which fourth column located in each mutated region and calculate the insert size of each read pairs. For each BAM/SAM file, calculate the mean and deviation of the insert size, and record the number of abnormal reads and normal reads for each structural variant.

#### Features based on the alignment information

We extract the numbers of incompletely mapped reads, split mapped reads, fully mapped reads, single mapped reads, unmapped reads, read directions, mapping qualities and affected reads as features based on the read alignment information.

**Fig. 3:**
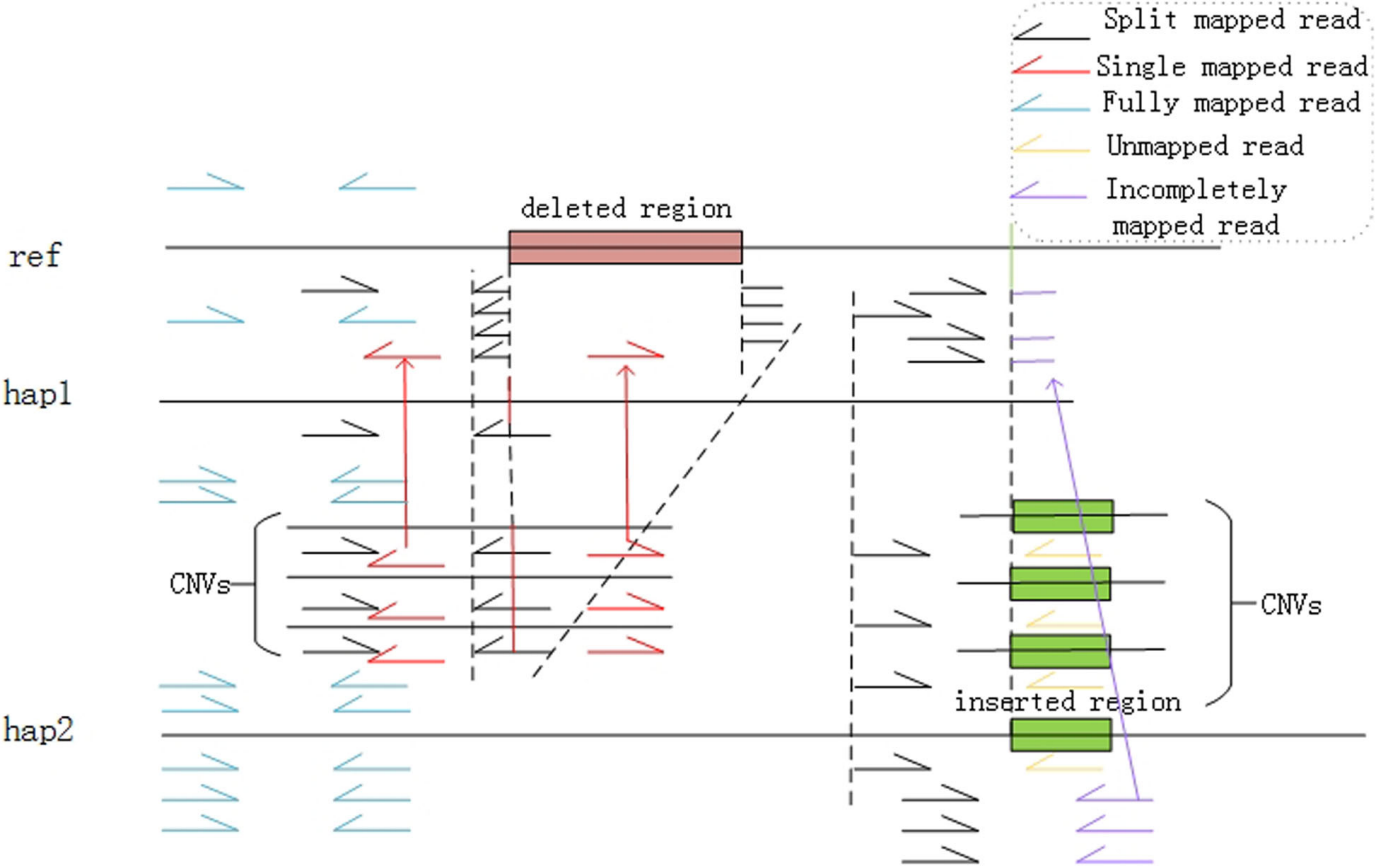
Features based on alignment information. The red rectangle indicates the deletion, and the green portion indicates the insertion variant

Figure 3 lists five situations that a read located in a mutated region may fall into, if the reads are perfectly mapped to the reference, we extract the perfectly mapped read pair as the fully mapped read and the other as the single mapped read. Conversely, if the reads cannot be perfectly mapped to the reference, we define the read which one segment mapped to the reference and the rest cannot as the incompletely mapped read, define the read separated into two parts and each part mapped to the reference successfully as split mapped read, and define the rest cannot perfectly mapped read as unmapped read. For a region harboring a structural variant, the incompletely mapped reads may be caused if a non-homologous insertion mutation occurs. If a homologous deletion or insertion occurs, it may cause the split mapped reads. If the non-homologous insertion is extremely long and a whole read fall into the insertion fragment, the read may be counted as an unmapped read. Similar to the previous description, when there exists a CNV, the ratio that the SV caused reads to normal reads will be increased if the CNV happens in the mutated haploytpe, while the ratio will be deceased if the CNV happens in the wild haplotype.

Furthermore, the two reads in a read pair is usually in the opposite direction and some structural variations may cause some reads to be reverse mapped to the reference, resulting the pair reads the same direction. If there are CNV and inversion, the reads harbor the inversion will show the same direction owing to the reverse complementarity, and the number of reads with same direction should not be ignored. Accordingly, we define the number of reads that clipping from 5’ during initial mapping and clipping from 3’ as two features. Moreover, the mapping quality is a measure of the confidence that a read actually comes from the position it is aligned to by the mapping algorithm [25]. Because of the structural variation, the mapping quality of read pairs in mutated regions varies greatly from genotypes and copy numbers, and the sum of the mapping qualities of reads in mutated region can be used as a feature to identify genotypes.

Same as the former features, we first extract the mutated region for each structural variant, select the row in the BAM/SAM file which fourth column located in each mutated region and extract the CIGAR value of the row, which can fully reflect the alignment signals. We then count the read pairs whose CIGAR value is equal to "100M" as fully mapped reads, the single read whose CIGAR is equal to "100M" as single mapped read. On the other hand, we count the read whose CIGAR value is not equal to "100M" as incompletely mapped reads. Thirdly, for each pair of reads (every two rows), we count the number of read pairs whose fourth column POS value are one in and the other out of the mutated region as split mapped reads. Fourthly, we extract the reads which located in the mutated region and cannot match to the reference using the *Samtools* command "./samtools view -bS -bf -h **.f.bam > **.sam" in the Linux terminal, so that the information file **.f.bam file (** is the user-defined file name) of the unmapped reads is obtained. Fifthly, we count the number of reads which located in the mutated region and the second column FLAG equals to "83" as the direction 1, count the number of reads which FLAG equals to "163" as the feature direction 2. Sixthly, we sum the corresponding fifth column MAPQ values of the reads that located in the mutated region in the BAM/SAM file for each SV, and record it as the value of Mapping qualities feature. The commands are all listed in the bat.sh file.

Last but not least, existing research found that there is a higher probability of base variation in the vicinity when structural variation occurs and different structural variations may lead to different probabilities of base variation [26]. Consequently, we extract the number of peripheral read pairs affected by structural variation as a feature and named it as the affected reads, which can reflect the existence characteristics of structural variation and facilitate the identification of multiple genotypes. We extract the second column and the eighth column, POS and End, from the VCF file, and set the [POS-length /10, End+length /10] as the variation vicinity range. We count the number of reads which fourth column POS value located in the variation vicinity range and recorded it as the affected reads value for each variant.

#### Features based on the read depth

Read depth refers to the number of reads mapped to a particular site or genomic region. Assuming that the sequence process is uniform, read depth follows a random (typically Poisson or modified Poisson) distribution [13] and the number of reads mapping to a genomic region is expected to be proportional to the ploidy that the region appears in the sequence sample. Compared with the normal region, the number of read depths in the mutated region will be reduced, while will be increased in the CNV region. Accordingly, the read depth may be a feature to distinguish five genotypes and we set the read depth(D) equals to the number of reads that located in the mutated region divide the *L*. Furthermore, we propose the weighted read depth as a new feature by weight the read depth with the coefficient *w*_*i*_.

1 $$ w_{i} = \frac{Q_{i}}{Q_{max}}.  $$

Where the *w*_*i*_ refers to the coefficient, *Q*_*i*_ refers to the MAPQ value of each mapped read pair, *Q*_*max*_ equals to the highest MAPQ value of the read in one BAM/SAM file. And then the weighted read depth can be calculated as:

2 $$ W_{RD} = \frac{\sum_{i = 0}^{n} wi}{L}  $$

The *n* refers to the number of reads that in the mutated region of each variant.

We further propose the extended weighted read depth feature to avoid considering the mutated region only and to make full use of the difference between the mutated region and the normal region. Comparing with the weighted read depth, we expand the mutated region from [Pos - 100, END + 100] to [Pos - 200, END + 200], based on the relationship between the reads and the *L*. The expanded weighted read depth can reflect the genotype characteristics of multiple variants from another point of view.

### Framework selection rationale

**Fig. 4:**
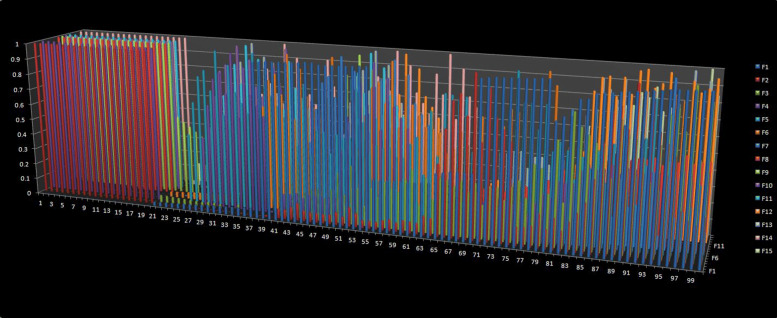
The distributions of the features values. The vertical axis represents the value of the features and the horizontal axis represents the structural variant calls. From 0 to 100, every 20 calls represent N, G1, G2, G3 and G4, respectively. F1 to F15 follows the same order as listed in Table 1. The figure indicated that the type N can be distinguished from types G1-4, but types G1, G2, G3, G4 are difficult to classify

**Fig. 5:**
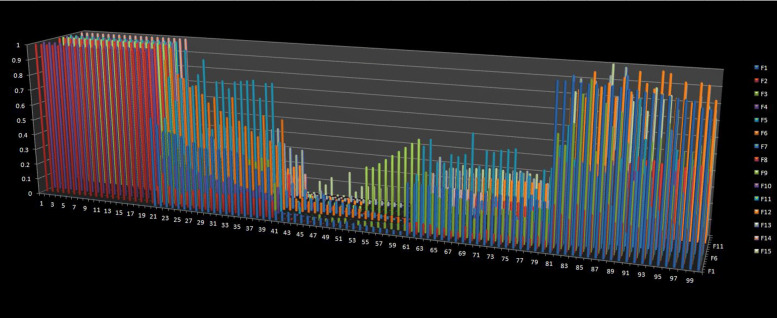
The features after the kernel function operation. The data presents five distinct linear categorizations after M-RVM kernel processing

To select the suitable machine learning framework, we sampled a random 1 Mbps region from the reference (version:hg19) and randomly planted 100 structural variants for each dataset. The type of the variants include insertion, deletion, inversion, complex indel and CNV. We created 20 candidates for each genotype and set the lengths of variations between 0.5 ∼5 kbps, while the lengths of CNVs between 1 ∼5 kbps. For each variant, we set an elevated region with 1000 bps longer than its own length and set the regional mutation rate to 0.01, some associated single nucleotide variants (SNVs) were planted in the preset elevated region and the background mutation rate was set to 0.0001. About one fourth inserted fragments of the complex indel came from nearby regions [27]. We set the read length to 100 bps, the distribution of insert sizes to follow the normal distribution of the 500 bps mean and 15 bps standard deviation, and the sequence error rate of reads sampling was considered as 0.005. We extracted the features and plotted them in Fig. 4, where the vertical axis represents the value of the features and the horizontal axis represents the structural variant calls. From 0 to 100, each of the 20 calls represent N, G1, G2, G3 and G4, respectively. The figure indicated that the type N can be distinguished from types G1-4, but types G1, G2, G3, G4 are difficult to classify. We tried a variety of machine learning models and found that the Multiclass Relevance Vector Machine (M-RVM) [28] works well for our datasets, and the five categories can be easily classified after the M-RVM kernel function transformation as shown in Fig. 5.

Compared with the popular methods and according to the description of the literatures, we suggest the M-RVM has the following advantages: 1. M-RVM adopts hierarchical Bayesian model structure and has the characteristics of flexible kernel function, overcoming the limitation that the selected kernel function must satisfy the Mercer condition, it is easier to find a suitable kernel function. 2. The membership probabilities of multiple classes output are realized by introducing Multi-probability likelihood functions. The M-RVM obtains probabilistic output and can directly output the prediction probability of each category. 3. M-RVM does not always depend on all feature data, which greatly reduces the calculation of kernel function, the test time and avoids over-fitting. 4.The M-RVM actually calculates the inner product of input vectors in feature space indirectly through the kernel mapping and does not need to be solved in high-dimensional space, which skillfully avoids the "Curse of Dimensionality" caused by the dimensions increase. We chose the M-RVM framework to deal with the linear and nonlinear datasets in higher dimension, and generated a more complex surface model in higher dimensional space according to data characteristics, so as to classify high-dimensional complex datasets non-linearly.

## Results

### Generating simulated datasets and testing model parameters

To evaluate the performance of the proposed method, we sampled a random 1 Mbps region from the reference (version:hg19), and then randomly planted 300 structural variants for each dataset. The type of the variants include insertion, deletion, inversion, complex indel and CNVs. Based on the distribution probability of CNV, we created 60 Type N calls (normal homozygous genotype without variants), 80 Type G1 calls (homozygous variant without CNV), 80 Type G2 candidates (heterozygous variant without CNV), 50 Type G3 candidates (heterozygous variant with CNV occurs in mutated haplotype) and 30 Type G4 calls (CNV occur in wild haplotype heterozygote). Set the lengths of variations between 0.5 ∼5 kbps, while the lengths of CNVs between 1 ∼5 kbps. For each variant, we set an elevated region with 1000 bps longer than its own length and set the regional mutation rate to 0.01, some associated SNVs were planted in the preset elevated region and the background mutation rate was set to 0.0001. About one fourth inserted fragments of the complex indel came from nearby regions [27]. We set the read length to 100 bps, the distribution of insert sizes to follow the normal distribution of the 500 bps mean and 15 bps standard deviation, and the sequence error rate of reads sampling was considered as 0.005.

**Fig. 6:**
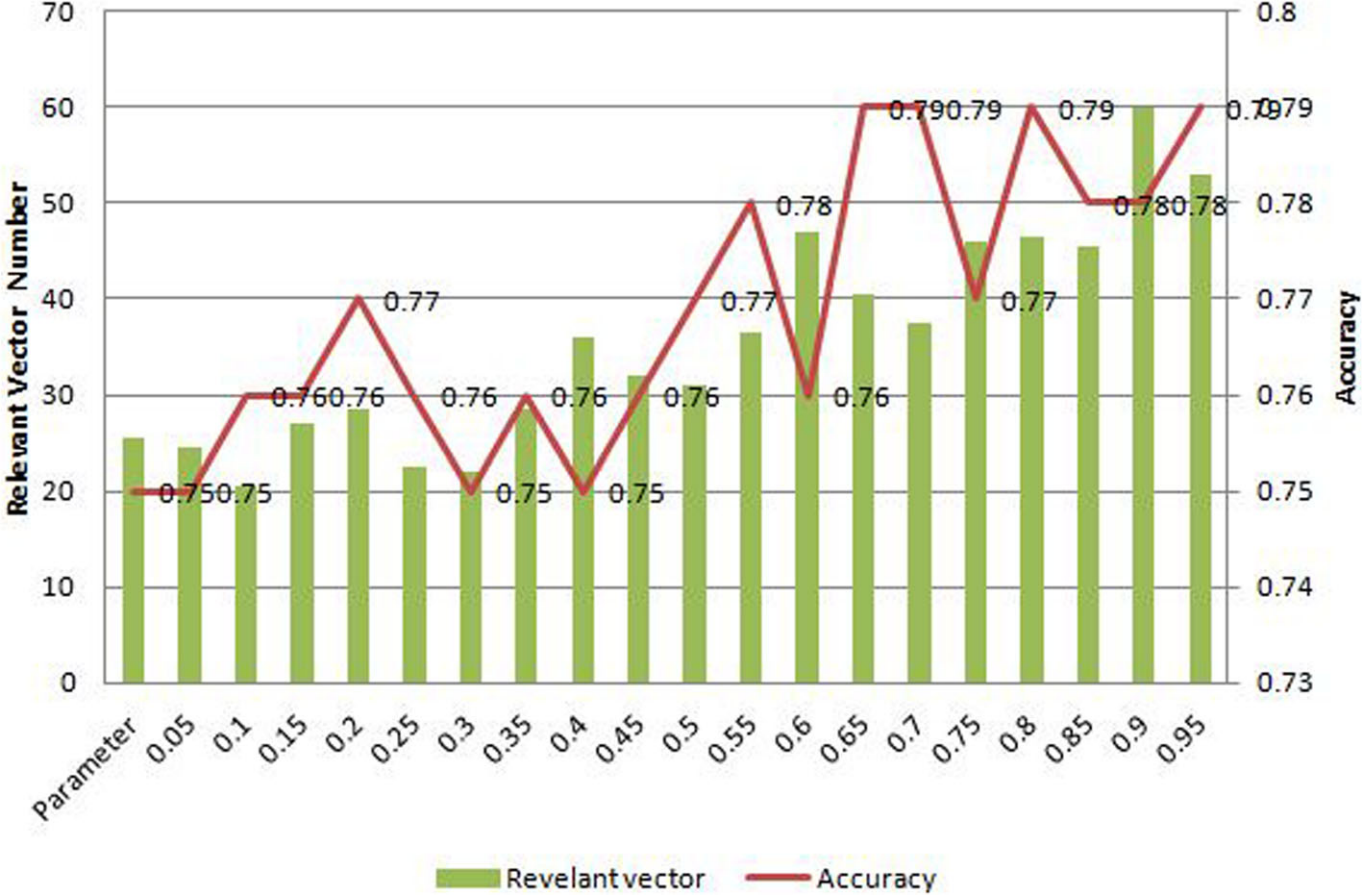
Kernel parameter selection. The vertical axis represents the accuracy and the number of relevant vector, while the horizontal axis represents the parameter. The results showed that when the kernel parameter is 0.7 (or 10), the accuracy is the highest and the number of relevant vectors is the least, which provide some enlightenment for parameter selection

The main evaluation index of the performance of the method are the accuracy and the number of relevant vectors, and are mainly affected by the kernel parameter. The accuracy refers to the ratio of the number of samples correctly classified by the classifier to the total number of samples for a given test dataset, and the relevant vector refers to the nonzero parameter corresponding point, which reflects the characteristics of the training data onto the reason that most of the parameters of the posterior distribution tend to zero and has nothing to do with forecast [29]. We first randomly selected five groups of coverage and copy number for parametric adjustment experiments and analyzed the influence of parameters on the accuracy of the method as shown in Fig. 6. The vertical axis represents the accuracy and the number of relevant vector, while the horizontal axis represents the parameter. The results showed that when the kernel parameter was 0.7 (or 10), the accuracy was the highest with the least relevant vectors, which provide some enlightenment for parameter selection.

### Comparing the performance on simulated dataset

To evaluate the performance of the proposed method comprehensively, we chose three existing methods to compare: 1) obtain the CNV region through the *Facets* [23] and then estimated the genotypes by 20%-80% rule (correct the interval by the copy-number), the snp.vcf.gz file required for the *Facets* was downloaded at https://www.ncbi.nlm.nih.gov/variation/docs/human_variation_vcf/. 2) *GATK* [24], default setting with HaplotypeCaller. 3) *Gindel* [13]. We compared the result at different copy numbers, and for each copy number configuration, we changed the coverage from 5 × to 20 ×. The accuracy of each experiment were listed in Table 2 and the third column represented the result from the proposed approach, the accuracy of *Gindel*, *Facets*, *GATK* were listed at column 4,5,6, respectively. Each result was an average of five repeated experiments.

**Table 2:** Performance comparison on simulated datasets

Copy Number	Coverage	**The proposed method**	*Gindel*	*Facets*	*GATK*
2	5 ×	0.8100	0.3054	0.4710	0.5328
	10 ×	0.8353	0.5342	0.5125	0.8157
	15 ×	0.8477	0.5728	0.5125	0.7697
	20 ×	0.8510	0.6175	0.5150	0.7039
3	5 ×	0.8142	0.4137	0.4713	0.7368
	10 ×	0.8195	0.4205	0.4912	0.4539
	15 ×	0.8168	0.4273	0.5124	0.8157
	20 ×	0.8195	0.4296	0.4625	0.8355
4	5 ×	0.8234	0.4296	0.5467	0.2560
	10 ×	0.8247	0.4201	0.5626	0.5942
	15 ×	0.8234	0.4268	0.5672	0.6714
	20 ×	0.8395	0.4336	0.5695	0.7101
5	5 ×	0.8313	0.4796	0.5573	0.3125
	10 ×	0.8358	0.4877	0.5630	0.6057
	15 ×	0.8356	0.4905	0.5645	0.6153
	20 ×	0.8413	0.4592	0.5680	0.7788

**Fig. 7:**
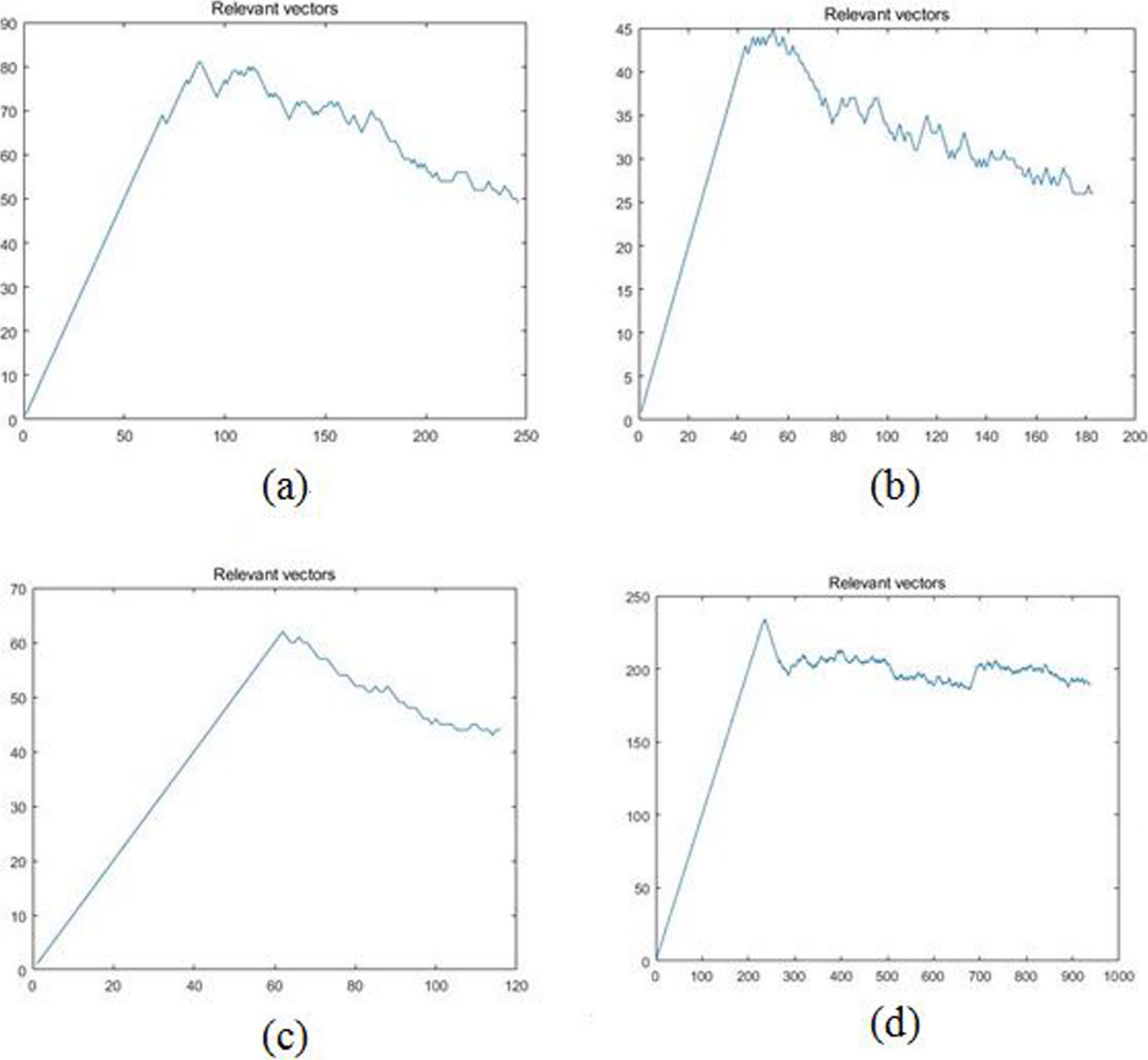
Relevant vectors during the simulation dataset training process. The horizontal axis represents the number of iterations, and the vertical axis represents the number of relevant vectors. Figure **a** - **d** are four randomly records in multiple experiments

The result demonstrated that the accuracy of our method was stable above 83% (±2%) while the classification accuracies of M-SVM, *Facets* and *GATK* were very low and unstable. The average of *M-SVM* was 45% and the range was about 30%, the mean of *Facets* was 52.79% and the range of it was 10.70%, of *GATK* were 63.8% and 57.95%. Specifically, as the coverage increased, the accuracies of these methods showed an increasing trend, and the methods decreased slightly as the copy number increased, which were all consistent with the theoretical principle. When the coverage and copy number changed, our method showed stable adaptability, strong robustness and maintained a high level. Furthermore, we visualized the variation of the relevant vectors in the iteration process as shown in Fig. 7, in which the horizontal axis represented the number of iterations, and the vertical axis represented the number of relevant vectors. The relevant vectors in the iterative learning process were obviously reduced after about 200 iterations, which showed that our model only needed a small number of relevant vectors and the data sparsity was reduced with the increase in iteration steps by the M-RVM approach. Consequently, our method could achieve higher model sparsity and shorter diagnosis time, the computational complexity was low on new sample input data diagnosis.

### Comparing the performance on real dataset

We obtained nine set of sequencing data from the Gene+ public database provided by Henan Cancer Hospital. All of the nine sets were the targeted sequencing reads with Gene+ 1021 panel. All the clinical information were removed, patients were numbered by a random target and all the germline variants were also removed before we obtained the data. The raw data has been already processed on the public data base, following the pipeline which the raw sequence read was mapped by *BWA-0.7.5a* [25] and *GATKMUTect*, *CNVkit* were used to detect the true structural variation information.

**Fig. 8:**
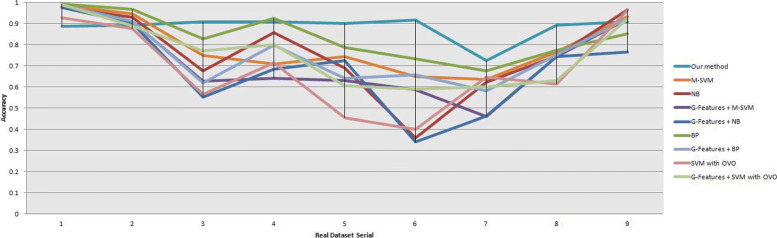
Methods comparison results on real dataset. The horizontal axis represents the real dataset serial, and the vertical axis represents the accuracy

**Fig. 9:**
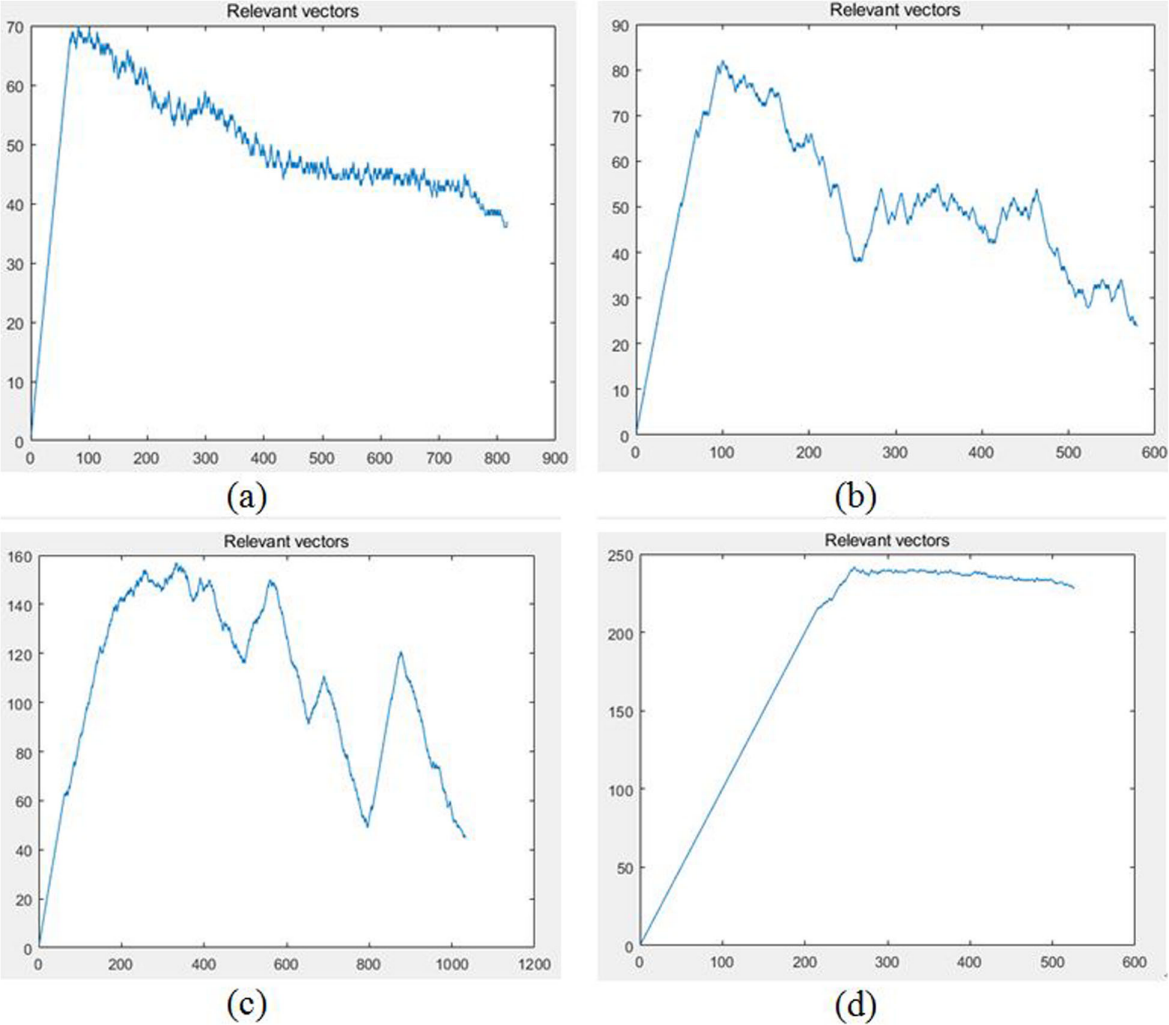
Relevant vectors during the real dataset training process. The horizontal axis represents the number of iterations, and the vertical axis represents the number of relevant vectors. Figure **a** - **d** are four randomly records in multiple experiments

We obtained the input SAM file and VCF file of the algorithm by processing the offline data and compared the proposed method with popular machine learning framework *SVM, Naïve Bayes, BP Neural Network* and *Lanrange-SVM with OVO multiple classification* as shown in Tables 3, 4 and Fig. 8. Because the popular method *Gindel* selected the discordant pair reads, split mapped read, read depth as features and was a binary classification method based on the SVM framework, we compared our method with multi-classification SVM and extra tested the *Gindel* features with the machine learning frameworks. The results showed that the proposed method can adapt well on real data and the experiment results were even better than simulation results because of the large sample size and high coverage of the real datasets. Compared with the popular algorithms, the average of our method accuracy was 88.20% (±15%), while the average of *M-SVM* was 79.17% (±20%), of *Naïve Bayes* was 75.85%(±40%), of *BP Neural Network* was 83.67%(±16%), of *Lanrange-SVM with OVO multiple classification* was 68.43%(±28%), of *Gindel* features + *M-SVM* was 72.31%(±26%), of *Gindel* features + *Naïve Bayes* was 68.40%(±34%), of *Gindel* features + *BP Neural Network* was 76.35%(±22%), and of *Gindel* features + *Lanrange-SVM with OVO multiple classification* was 75.57%(±23%), which indicated that our method maintained higher accuracy and stability. We also visualized the change of the relevant vectors in the iteration as shown in Fig. 9, which confirmed that our method maintained high model sparsity and short diagnosis time. These indicated that our method has the advantages of accuracy and computation, and can be well applied to clinical practice.

**Table 3:** Performance comparison on real datasets

	Coverage	InsertSize	The proposed method	*G-Features+M-SVM*	*G-Features+NB*	*M-SVM*	*NB*
1	472 ×	216	0.8875	0.9851	0.9777	0.9880	0.9777
2	396 ×	213	0.8917	0.9098	0.9031	0.9469	0.9317
3	394 ×	223	0.9083	0.6265	0.5513	0.7493	0.6766
4	291 ×	175	0.9082	0.6403	0.6839	0.7089	0.8582
5	448 ×	214	0.9000	0.6299	0.7254	0.7444	0.6886
6	460 ×	210	0.9167	0.5881	0.3415	0.6502	0.3589
7	402 ×	202	0.7258	0.4603	0.4632	0.6361	0.6217
8	422 ×	207	0.8917	0.7444	0.7439	0.7660	0.7469
9	369 ×	215	0.9083	0.9234	0.7667	0.9336	0.9669
Average			0.8820	0.7230	0.6840	0.7914	0.7585
Range			0.1909	0.5248	0.6362	0.3519	0.6188

In practice, we adopted the multi-classification support vector machine (M-SVM) as a plus version because the *Gindel* is a binary classification method based on support vector machine (SVM), it will treat the other three genotypes as classification errors and lead to low accuracy when applied directly

**Table 4:** Performance comparison on real datasets-2

	**The proposed method**	*BP*	*G-Features+BP*	*SVM with OVO*	*G-Feature+SVM with OVO*
1	0.8875	0.9929	0.9868	0.9279	0.9897
2	0.8917	0.9677	0.9122	0.8753	0.8910
3	0.9083	0.8270	0.6190	0.5653	0.7699
4	0.9082	0.9251	0.7983	0.7131	0.8018
5	0.9000	0.7870	0.6417	0.4540	0.6057
6	0.9167	0.7324	0.6566	0.4017	0.5884
7	0.7258	0.6752	0.5807	0.6455	0.6016
8	0.8917	0.7724	0.7513	0.6131	0.6302
9	0.9083	0.8513	0.9250	0.9633	0.9230
Average	0.8820	0.8367	0.7635	0.6843	0.7557
Range	0.1909	0.3177	0.4061	0.5616	0.4013

## Discussion

### Performance tests with considering the low complexity region

There is a research topic in the genome called the low complexity region (LCR), which contains one or more genes, pseudogenes, gene fragments, retroviral sequences and gene regulatory regions, usually located in telomeres and telomeres. Its repeats create ambiguities in alignment and in genome assembly, which, in turn, can produce errors when interpreting results. Repeat that is sufficiently divergent do not present problems, the LCR defined a repeat as a sequence that is at least 100bps in length, occurs two or more times in the genome and exhibits >97% identity to at least one other copy of itself. This definition excludes many repetitive sequences, but it includes those present the principal computational challenges [30].

To better test the performance of the proposed method, we tested the method on the simulated dataset considering the low complexity region. We downloaded the bed file of LCR from ENCODE Project (Encyclopedia of DNA Elements) website, and inserted the recorded LCR information into the corresponding region fragments of chromosome hg19 and constructed reference containing LCR. The simulation data which meet the requirements of the existing popular literature [31] was constructed through the structural variation and CNV planting. The results were shown in the Table 5, where the average of the experiment was 81.51%, which indicated that our method can effectively deal with the LCR impact.

**Table 5:** Performance test considering of the low complexity region

Copy Number	Coverage	Accuracy	Parameter number
2	5 ×	0.7800	8
	10 ×	0.8153	14
	15 ×	0.8277	20
	20 ×	0.8310	35
3	5 ×	0.7942	10
	10 ×	0.8177	15
	15 ×	0.8168	22
	20 ×	0.8195	37
4	5 ×	0.7934	12
	10 ×	0.8147	24
	15 ×	0.8285	32
	20 ×	0.8295	37
5	5 ×	0.8113	14
	10 ×	0.8258	17
	15 ×	0.8256	24
	20 ×	0.8113	37

### Result discussion and further study plan

We try to explore the loss of accuracy of our method, which can be discussed from three aspects. First, the reason why that the classification accuracy of the simulation data is lower than the real data; Second, the reason for the accuracy loss of the real data; Third, the reason why the accuracy of real data fluctuates. Based on the experiment results, the accuracy of the simulation data is 83% on average, and the mean accuracy of the real data is 88.20%. We analyzed the data in detail and found that the reason for this phenomenon is that the sample size and the coverage of the simulation data is small, which we set as 300 and 5 ∼20×. In comparison, the scale of real data is thousands of times of simulation data, and the coverage is dozens of times of simulation data, which has better classification accuracy and model training advantage consequently.

Secondly, we try to explain the accuracy loss of the real datasets. On the one hand, the accuracy of the machine learning model is not expected to be 100%. The more classification categories, the higher the probability of accuracy loss will be. Moreover, we observed the output probability matrix of the model and found that there are a small number of samples have the equal calculated probabilities of five genotypes, our method directly considered these samples as error classifications. We counted the number of the sample of equal probability results in the real datasets as shown in Table 6, and found that the samples of equal probability were 1% of the total samples and the range was 2.04%. We set the equal probability samples as invalid samples and recalculated the ratio of the correct classified samples of all the valid samples. The mean accuracy was 0.89% higher than the original accuracy, and the range growth was 1.85%.

**Table 6:** Result discussion

	Accuracy	Sample capacity	Invalid sample	**Accuracy of valid sample**
1	0.8875	960	23	0.9093
2	0.8917	4800	35	0.8983
3	0.9083	2624	23	0.9163
4	0.9082	3990	27	0.9144
5	0.9000	6810	30	0.9040
6	0.9167	5470	25	0.9210
7	0.7258	1047	23	0.7421
8	0.8917	2024	21	0.9011
9	0.9083	6950	25	0.9116

In addition, we also found some problems worthy of further study. When dealing with real data, there were not many samples of genotype markers for copy number loci (which is the reason that we only did real data experiments for nine patients). One reason is that the sequence company lack of this awareness, for the mechanism of individual copy number is relatively clear, and other unclear ones are not necessary for labeling. Another reason is costing considerations. Motivated by these, we want to explore the use of semi-supervised machine learning framework to learn only a small number of labeled data and train a general model for classification. The idea is being explored in another article.

## Conclusion

In this article, we focused on the genotyping of structural variations with copy number variant, and proposed a machine learning method based on M-RVM. CNV is widely exists in cancer genome, which causes the misjudgment of structural variation genotyping by existing methods and greatly reduces the accuracy of processing cancer data. The correctly distinguish the position of CNV from the structural variation genotypes is necessary. Accordingly, we transformed the problem of genotyping into a multi-classification problem and 15 features were carefully selected as input on the basis of observation and practice. Based on the data analysis of features, we chose M-RVM framework, which can efficiently deal with the problem of low-dimensional linear inseparability, achieve efficient classification results and output the result of genotyping with the greatest possibility. We tested the performance of this method and compared it with existing popular genotype method *Gindel*, *GATK*, *Facets* and four commonly used machine learning methods: *SVM, Naïve Bayes, BP Neural Network* and *Lanrange-SVM with OVO multiple classification*. The results showed that the proposed method significantly improved the accuracy of structural variations genotyping and the mean recognition rate of this method was obviously higher than other classification methods under the same conditions. In conclusion, our method is stable, reliable, robust, useful for genotyping and downstream operation, and has good response to coverage and copy number, which anticipates a wider usage in cancer clinical sequence.

## Abbreviations

CNV: Copy number variant
SVs: Structural variations
VAF: Variant allelic frequency
SAM: Standard alignment/map
BAM: Binary alignment/map
VCF: Variant call format
SNV: Single nucleotide variant
M-RVM: Multiclass relevance vector machine
bp: Base pair
M-SVM: Multiclass support vector machine
BP Neural Network: Back propagation neural network
NB: Naïve Bayes
OVO: One-versus-one
LCR: Low complexity region
